# Molecular biomarkers in cardiac hypertrophy

**DOI:** 10.1111/jcmm.14129

**Published:** 2019-01-16

**Authors:** Liu Zhu, Chao Li, Qiang Liu, Weiting Xu, Xiang Zhou

**Affiliations:** ^1^ Department of Cardiology The Second Affiliated Hospital of Soochow University Suzhou China; ^2^ Department of Cardiology Fuwai Hospital Chinese Academy of Medical Sciences Shenzhen China

**Keywords:** cardiac hypertrophy, micro‐RNAs, molecular biomarkers

## Abstract

Cardiac hypertrophy is characterized by an increase in myocyte size in the absence of cell division. This condition is thought to be an adaptive response to cardiac wall stress resulting from the enhanced cardiac afterload. The pathogenesis of heart dysfunction, which is one of the primary causes of morbidity and mortality in elderly people, is often associated with myocardial remodelling caused by cardiac hypertrophy. In order to well understand the potential mechanisms, we described the molecules involved in the development and progression of myocardial hypertrophy. Increasing evidence has indicated that micro‐RNAs are involved in the pathogenesis of cardiac hypertrophy. In addition, molecular biomarkers including vascular endothelial growth factor B, NAD‐dependent deacetylase sirtuin‐3, growth/differentiation factor 15 and glycoprotein 130, also play important roles in the development of myocardial hypertrophy. Knowing the regulatory mechanisms of these biomarkers in the heart may help identify new molecular targets for the treatment of cardiac hypertrophy.

## 1 INTRODUCTION

Myocardial hypertrophy is characterized by the thickening of heart muscles without an obvious cause and found to be involved in several pathological conditions, including hypertension, vascular disease and chronic heart failure.^1^ Myocardial hypertrophy was first described by Donald Teare in 1958.^2^, ^3^ The results of epidemiological studies indicate that the increased incidence and prevalence of heart dysfunction is one of the primary causes of morbidity and mortality in elderly people. The pathogenesis of heart dysfunction is multifactorial and often associated with cardiac remodelling as a result of cardiac myocyte hypertrophy.^4^, ^5^, ^6^ Myocardial hypertrophy is a response to pressure or volume overload whereas chronic left ventricle hypertrophy is primarily associated with chronic heart dysfunction. In addition, myocardial hypertrophy is thought to be a maladaptive process that leads to the fatal gene program and pro‐hypertrophic signalling pathways.^7^, ^8^, ^9^ In the adult heart, the progression of myocardial hypertrophy follows the signals which are stimulated on the cell surface and then transmitted through channels or receptors.^10^ Therefore, we describe molecules involved in the development and progression of myocardial hypertrophy to well understand underlying mechanisms.

## 2 MICRO‐RNAS

Micro‐RNAs are non‐coding post‐transcriptional regulatory RNAs that play a key role in regulating mRNA expression and heart diseases.^11^ Recent studies indicated that changes in the expression levels of micro‐RNAs may contribute to the development of cardiac hypertrophy.^12^

### 2.1 MiR‐96

MiR‐96 is a micro‐RNA involved in many diseases caused by impaired cell proliferation.^13^, ^14^, ^15^, ^16^ It has been confirmed that the mammalian target of rapamycin (mTOR) signalling pathway promotes the onset and progression of cardiac growth.^17^, ^18^, ^19^, ^20^MiR‐96 is negatively correlated with mTOR and may prevent myocardial hypertrophy by inhibiting mTOR.^20^ On the other hand, miR‐96 was found to inhibit cardiac hypertrophy by targeting growth factor receptor‐bound 2 which is a negative regulator of cardiac hypertrophy.^21^ Whether it may offer a new therapeutic strategy for cardiac hypertrophy is still controversial.

### 2.2 MiR‐30

Several studies have demonstrated that the miR‐30 family micro‐RNAs are involved in the development and progression of tumours and other diseases, including those in the circulatory, genital, respiratory, nervous, alimentary and genital systems. MiR‐30 members play a vital role in cell differentiation, cellular senescence, adipogenesis and drug metabolism.^22^, ^23^, ^24^, ^25^, ^26^, ^27^, ^28^, ^29^, ^30^ Duister et al. found that miR‐30 could trigger myocardial matrix remodelling by regulating the connective tissue growth factor, and the expression of miR‐30 was significantly decreased in mice with hypertrophic cardiomyopathy.^31^ Reversely associated with p53 up‐regulation, miR‐30 family members are found to inhibit mitochondrial fission and the consequent cardiac hypertrophy related apoptosis.^32^ Furthermore, cardiac hypertrophy due to chronic alcohol intake in mice was correlated with a lower expression of miR‐30a.^33^ Another study demonstrated that the expression of beclin‐1 was up‐regulated in cardiomyocytes treated with miR‐30a inhibitor, and the up‐regulation was reversed in cardiomyocytes treated with miR‐30a mimic.^34^ This study further suggested that the activation of autophagy was enhanced by treating angiotensin II (Ang II)‐induced hypertrophic cardiomyocytes with miR‐30a inhibitor, and this activation was reversed in cells treated with miR‐30a mimic. These results suggest that beclin‐1 can be bound to miR‐30a as a target gene for miR‐30a. Therefore, miR‐30a is a potential diagnostic and therapeutic marker for hypertrophic cardiomyopathy.

### 2.3 MiR‐34

In mammals, the miR‐34 miRNA precursor can produce three major mature miRNAs. In contrast to other members of this family, the miR‐34 was identified in silico and later confirmed by experiment. In the cell cytoplasm, the precursor miRNA stem‐loop is excised, and the primary miR‐34 mature sequence is removed from the 5′ arm of the hairpin.^35^, ^36^, ^37^ MiR‐34a is a multifunctional regulatory factor that participates in apoptosis,^38^ cell senescence,^39^ cell proliferation^40^ and cell division^41^ by enhancing or reducing the expression of its target genes. Studies demonstrated that miR‐34a is a component of the p53 tumour suppressor network, suggesting that this mRNA participates in cancer development and progression.^42^ The expression of miR‐34a varies according to the pathological condition. In myocardial infarction^43^ and ageing heart,^44^ the expression of miR‐34a is increased, whereas in most cancers, including bladder and lung cancer, miR‐34a expression is usually decreased.^45^, ^46^ Moreover, as a result of transverse aortic constriction, pressure overload leads to changes in miR‐34a expression in different pathological stages of cardiac remodelling,^47^ such that the expression is decreased in the hypertrophic stage of myocardial remodelling but increased in the heart failure stage. Another study found that miR‐34a extended *Caenorhabditis elegans* lifespan by inhibiting the activation of ATG9A‐mediated autophagy.^48^ It is known that Ang II can regulate cardiac hypertrophy and cardiomyocyte autophagy.^49^ MiR‐34a also can regulate Ang II‐induced cardiomyocyte hypertrophy by directly inhibiting ATG9A expression and autophagic activity.^47^ It has been found that therapeutic inhibition of the miR‐34 family attenuates pathological cardiac remodelling and improves heart function in mice.^50^ Therefore, the results of these studies may help identify prospective therapeutic targets for controlling cardiac hypertrophy.

### 2.4 MiR‐181

The miR‐181 miRNA precursor is a small non‐coding RNA molecule that can generate four mature products: miR‐181a, miR‐181b, miR‐181c and miR‐181d. These products can regulate post‐transcriptional gene expression by binding to target mRNAs. MiR‐181 has been found in a large number of species, including humans, zebrafish and rats. Human miR‐181 has been found in bone marrow, retina and vascular development.^51^, ^53^ Furthermore, the expression of miR‐181b was up‐regulated in the blood of patients with myocardial hypertrophy, suggesting that miR‐181b might play a role in both disease pathology and progression.^54^ Myocardial hypertrophy triggered by stimulation of primary myocardial cells with PE presented a negative correlation between miR‐181b and PKG 1.^54^ The results suggest that miR‐181b is a promising molecular marker for the clinical diagnosis and treatment of cardiac hypertrophy.

### 2.5 Other micro‐RNAs

MiR‐378 inhibited caspase‐3 expression in cardiomyocytes and attenuated ischaemic injury, and miR‐199a might be a potential therapeutic target for cardiac hypertrophy or heart failure. It has demonstrated that miR‐185 can suppress the progression of cardiac hypertrophy by reducing ET‐1‐induced hypertrophic responses^55^ whereas miR‐19a/b positively regulates cardiac hypertrophy by enhancing these responses.^56^ Lee et al. found that miR‐374 inhibited the cardiac hypertrophy regression pathway by targeting vascular endothelial growth factor receptor 1 (VEGFR‐1) and PKG‐1.^57^ It is also reported that miR‐9 and miR‐98 were closely related to the development of myocardial hypertrophy.^58^ Furthermore, the decrease in miR‐133 might reduce endothelin‐1‐ and norepinephrine‐induced myocardial hypertrophy.^59^

## 3 VEGF‐B

VEGF‐B is a secretory protein from the VEGF family. VEGF‐A is the best known member of this family, and other members include VEGF‐C and PIGF.^60^, ^61^ In humans, VEGF‐B is highly expressed in metabolically active tissues, including fat, heart and skeletal muscle. VEGF‐B has two isoforms produced by alternative splicing, VEGF‐B167 and VEGF‐B186, and both isoforms bind to VEGFR‐1 and neuropilin‐1.^62^, ^63^ In contrast to other members of the family, VEGF‐B is not a major angiogenesis‐inducing factor; however, it can enhance neovascularization under pathological conditions.^63^ It has been reported that VEGF‐B helps maintain newly formed blood vessels during pathological conditions.^64^ Furthermore, VEGF‐B regulates the uptake and transport of fatty acids in the endothelium of heart and skeletal muscle.^65^, ^66^ Karpanen et al. found that up‐regulating the expression of VEGF‐B in mouse heart changed lipid metabolism in myocardial cells and led to myocardial hypertrophy.^67^ Therefore, VEGF‐B is a potential target for the diagnosis and treatment of myocardial hypertrophy.

## 4 SIRT3

NAD‐dependent deacetylase sirtuin‐3 (SIRT3) is a member of the mammalian sirtuin family.^68^ Evidence indicates that SIRT3, as a soluble protein, is located in the mitochondrial matrix and contains a mitochondrial processing peptide at the N‐terminus. Up‐regulating the expression of SIRT3 in vitro inhibits the production of reactive oxygen species and promotes respiration. In white and brown adipose tissue, fasting increases the expression of SIRT3, and the increased expression of SIRT3 in brown adipocytes enhances the expression of UCP1 and PGC‐1α, demonstrating that SIRT3 has a role in adaptive thermogenesis in brown adipose tissue. Furthermore, SIRT3 reduces cardiac hypertrophy by decreasing ROS production.^69^, ^70^, ^71^ SIRT3 augments Foxo3a‐dependent antioxidant defense and further blocks the cardiac hypertrophic response.^72^

## 5 GDF15

The growth/differentiation factor 15 (GDF15) was first identified as macrophage inhibitory cytokine‐1.^73^ It is a member of the transforming growth factor‐beta superfamily. The expression of GDF‐15 in humans is low in most organs and increased by injury to different organs, including heart, kidney, liver and lung.^74^, ^75^, ^76^ GDF‐15 regulates inflammatory pathways and participates in several biological processes, including cellular repair and regulation of apoptosis and cell growth, as well as in cardiovascular and neoplastic disorders.^74^, ^77^, ^78^ GDF‐15 is a powerful prognostic marker in patients with heart disease and cancer.^79^ Recently, a growing number of studies have focused on the association between GDF‐15 and cardiovascular diseases. It has been proven that GDF‐15 is a good prognostic marker for coronary heart disease and heart failure,^80^, ^81^, ^82^, ^83^, ^84^, ^85^, ^86^, ^87^, ^88^, ^89^ and participates in cardiac remodelling caused by primary hypertension, hypertrophic cardiomyopathy and ischaemic heart diseases.^89^, ^90^, ^91^, ^92^, ^93^ Furthermore, the increased expression of GDF‐15 inhibits norepinephrine‐induced myocardial hypertrophy by decreasing EGF receptor transactivation after norepinephrine stimulation.^93^ These results indicate that GDF‐15 may be a new target for treating myocardial hypertrophy.

## 6 GP130

The transmembrane protein glycoprotein 130 (Gp130) is one of the first cytokine receptors to be identified. Gp130 is a signal‐transducing receptor element that binds to the interleukin 6 receptor. Moreover, this molecule is present in several receptors, including IL‐6, IL‐11, ciliary neurotrophic factor and leukaemia inhibitory factor receptors.^94^, ^95^, ^96^ Gp130 is strongly expressed in almost all tissues.^96^ Nonetheless, the physiological functions of gp130 are not completed explained. A previous study has shown that gp130 has a physiological role in cardiomyocyte regulation, whereas a pathological consequence leading to cardiac hypertrophy will happen after being overstimulated of gp130.^97^

## 7 CAMK II

Ca^2+^/calmodulin‐dependent protein kinase II (CaMKII) is a serine/threonine‐specific protein kinase regulated by the Ca^2+^/calmodulin complex. CaMKII participates in many signalling pathways and is considered a key mediator of learning and memory.^98^ In addition, CaMKII contributes to Ca^2+^ reuptake and homeostasis in cardiomyocytes,^99^ CD8 T‐cell activation,^100^ positive T‐cell selection^101^ and epithelial transport of chloride.^102^ CaMKII consists of four different isoforms: CaMKIIα, CaMKIIβ, CaMKIIδ and CaMKIIγ. CaMKIIδ is the main cardiac CaMKII isoform, and CaMKIIγ is also expressed in the heart. The expression and activity of CaMKII are enhanced in patients having heart failure.^103^, ^104^ Furthermore, the transgenic overexpression of CaMKII induced cardiac hypertrophy and dilated cardiomyopathy.^105^, ^106^ Structural heart disease may be prevented by CaMKII inhibitors.^107^, ^108^ Moreover, cardiac remodelling was attenuated in mice with global deletion of CaMKIIδ.^109^, ^110^ Backs et al. demonstrated that CaMKII could inhibit cardiac hypertrophy via crosstalk with calcineurin.^110^ Calcineurin has no function in maladaptive cardiac remodelling in the absence of CaMKII signals.^111^

## 8 CIC‐3

Chloride channel‐3 (ClC‐3) is a member of the ClC gene family and has been suggested to be a molecular candidate of native volume‐sensitive outwardly rectifying anion channels (VSOAC) in certain mammalian cell types, such as vascular smooth muscle cells and cardiac myocytes.^112^, ^113^ In ClC‐3 global knockout mice, the rest of VSOAC in cardiac myocytes caused extensive compensatory changes and altered properties in membrane protein expression.^114^ It is found that inactivation of ClC‐3 gene produced myocardial hypertrophy and heart failure.^114^ Furthermore, ClC‐3 is a key molecular of native VSOAC in mammalian heart and plays a crucial role in preventing myocardial hypertrophy.^114^

## 9 OTHER MOLECULES

The expression of adhesion molecules including CD11a, CD11b and CD11c was increased in rats with myocardial hypertrophy.^115^ Walsh reported that the inhibition of stress‐induced activin A/Smad2 signalling triggered the expression of follistatin‐like in hypertrophied cardiac myocytes.^116^ Follistatin‐like 3 is a stress‐induced regulator of cardiac hypertrophy and may regulate myocyte size via Smad signalling.^116^ Moreover, the expression of N‐cadherin was up‐regulated in myocardial tissues in rats. Although these findings are based on animal experimentation, it provides additional motivation for researchers to explore the functions of these biomarkers in regulating myocardial hypertrophy in human beings, either in modulating physiological environment or developing molecular target drugs.

## 10 CONCLUSIONS

Myocardial hypertrophy is adaptive response to cardiac wall stress resulting from the enhanced cardiac load. The main feature of myocardial hypertrophy is the embryonic genes up‐regulation, protein synthesis increases and cell volume expansion. The predominant histopathological characteristics are expanded cell space, increased intercellular muscle fibres and myocardial fibrosis and dysfunction. In this review, we list several micro‐RNAs and other molecular biomarkers involved in the pathogenesis of cardiac hypertrophy (Table 1). Knowing the regulatory mechanisms of these biomarkers may help identify new molecular targets for the treatment of cardiac hypertrophy.

**Table 1:** Molecular biomarkers involved in cardiac hypertrophy

Biomarkers	Characteristics
micro‐RNAs	
MiR‐96	Inhibit mTOR1 and reduce growth factor receptor‐bound 2 expression^20^, ^21^
MiR‐30	Inhibit cardiac hypertrophy related apoptosis^32^
MiR‐34	Up‐regulated in the heart in response to stress; improve cardiac function^50^
MiR‐181	Up‐regulated in myocardial hypertrophy^54^
VEGF‐B	Up‐regulated in lipid metabolism in myocardial cells^65^, ^66^; lead to myocardial hypertrophy^67^
SIRT3	Block cardiac hypertrophic response by augmenting Foxo3a‐dependent antioxidant defense^72^
GDF15	Inhibit norepinephrine‐induced myocardial hypertrophy^93^
GP130	Lead to cardiac hypertrophy by activating its expression^97^
CaMK II	Increased in hypertrophied myocardium and related to cardiac hypertrophy^105^, ^106^, ^107^, ^108^, ^109^, ^110^, ^111^
CIC‐3	Associated with myocardial hypertrophy and heart failure^114^

CaMK II, calmodulin‐dependent protein kinase II; ClC‐3, chloride channel‐3; GDF15, growth/differentiation factor 15; GP130, glycoprotein 130; SIRT3, NAD‐dependent deacetylase sirtuin‐3; VEGF‐B, vascular endothelial growth factor B; mTOR1, mammalian target of rapamycin 1.

## CONFLICT OF INTEREST

The authors declare that there are no conflicts of interest.
